# Comment on: “Prevalence and determinants of poor sleep quality among patients of diabetes mellitus: a tertiary hospital-based cross-sectional study”

**DOI:** 10.1097/MS9.0000000000004157

**Published:** 2025-10-17

**Authors:** Hamza Yousuf Ibrahim, Saaz Kumar, Ghansham Das, Tirath Patel

**Affiliations:** a Department of Medicine, Jinnah Medical and Dental College, Karachi, Pakistan; b Department of Surgery, Trinity Medical Sciences University School of Medicine, Kingstown, Saint Vincent and the Grenadines

**Keywords:** Critical appraisal, Metformin, Research methodology, Sleep quality, Type 2 diabetes mellitus

## Abstract

Hamayal *et al* found a significant correlation between metformin exposure and poor sleep quality in patients with diabetes, highlighting a significant clinical issue. However, the study’s validity, reproducibility, and generalizability are limited by methodological limitations. Causal inference is not possible due to the cross-sectional and single-center design, convenience sampling, and selection bias. The use of a self-report instrument, such as the Pittsburgh Sleep Quality Index, increases measurement and recollection bias and risks false classification of sleep disturbance. The relationship between metformin exposure and sleep quality is prone to residual confounding by diabetes duration, glycemic control, comorbidity burden, medication use, depressive symptoms, and socioeconomic factors. Reverse causality is also possible, as disrupted sleep may aggravate glycemic indices. Future research should adopt a prospective, multicentric design, objective measures of sleep, standardized definitions of case and outcome, and examine putative mechanisms involving antidiabetic interventions and sleep physiology.

*To the Editor*,

We read with interest the recently published article titled “Prevalence and determinants of poor sleep quality among patients of diabetes mellitus: a tertiary hospital-based cross-sectional study,” by Hamayal *et al*, published in Annals of Medicine & Surgery in July 2025^[1]^. In this study, the authors draw attention to an important yet frequently neglected aspect of diabetes management: sleep quality. Although the finding that 82% of patients experience poor sleep is noteworthy, several methodological considerations merit clarification.

First, the reported association between metformin use and poor sleep quality should be interpreted cautiously. Prior evidence supporting this relationship remains mixed. Some studies found that metformin treatment significantly increased actual sleep time (*P*= 0.01) and sleep efficiency (*P*= 0.04) in people with metabolic syndrome, suggesting that metformin may improve sleep quality as an add-on treatment for sleep disorders^[2]^, while a few case reports describe insomnia or vivid dreams in patients using metformin^[3,4]^. In the present study, confounding factors such as longer disease duration, comorbidities, or polypharmacy were not fully controlled. These may explain why metformin users appeared to have worse sleep, rather than reflecting an actual drug effect.

Secondly, the cross-sectional design limits causal inference. Although the authors report that uncontrolled diabetes and comorbidities predict poor sleep, it is equally possible that poor sleep worsens metabolic control by impairing insulin sensitivity, increasing counter-regulatory hormones, and predisposing to hyperglycemia and vascular complications^[5]^. Thus, this relationship is likely a bidirectional link, emphasizing the value of longitudinal research to determine temporal causality.

Thirdly, in this study, sleep quality was measured only through the self-reported Pittsburgh Sleep Quality Index (PSQI). Despite its validation worldwide, this tool remains subjective and prone to both reporting and recall biases. Studies have highlighted that subjective sleep scores frequently differ from objective measurements obtained through actigraphy or polysomnography^[6]^. Including such objective methods in future studies would allow for a more accurate evaluation of sleep disturbances and their link with glycemic control.

Finally, the authors recommend incorporating “sleep quality-oriented guidelines” into diabetes management protocols. While important in principle, over-generalizing from a single-center, convenience-sampled study may risk premature policy implications. Larger multicenter studies with a prospective design, objective sleep assessments, and thorough adjustment for potential confounders are necessary to establish the evidence needed for incorporation into clinical guidelines.

In summary, the study provides valuable preliminary evidence that underscores the importance of addressing sleep in diabetes care. We encourage the authors to expand on this work through prospective, multicenter studies that utilize objective sleep assessments and more comprehensive adjustments for confounders.

This manuscript is made compliant with the TITAN checklist to ensure transparency in the reporting of Artificial Intelligence^[7]^.

## Data Availability

No new dataset was generated during the study.

## Research registration unique identifying number (UIN)

Not required because the study is a letter to the editor.
